# A new outlier detection method for spherical data

**DOI:** 10.1371/journal.pone.0273144

**Published:** 2022-08-24

**Authors:** Adzhar Rambli, Ibrahim Bin Mohamed, Abdul Ghapor Hussin

**Affiliations:** 1 Centre of Statistics & Decision Science Studies, Faculty of Computer and Mathematical Sciences, Universiti Teknologi MARA, Shah Alam, Selangor, Malaysia; 2 Institute of Mathematical Sciences, University of Malaya, Kuala Lumpur, Malaysia; 3 Faculty of Defence Science and Technology, National Defence University of Malaysia, Kuala Lumpur, Malaysia; Yunnan University of Finance and Economics, CHINA

## Abstract

In this study, we propose a new method to detect outlying observations in spherical data. The method is based on the *k*-nearest neighbours distance theory. The proposed method is a good alternative to the existing tests of discordancy for detecting outliers in spherical data. In addition, the new method can be generalized to identify a patch of outliers in the data. We obtain the cut-off points and investigate the performance of the test statistic via simulation. The proposed test performs well in detecting a single and a patch of outliers in spherical data. As an illustration, we apply the method on an eye data set.

## Introduction

Spherical data are concerned with directions in three dimensions. They may arise in many areas of scientific experimentation such as biological, geological and environmental sciences. For example, the wind direction measured by two different equipments (see [1]) or the altitudes of the moon and the sun observed at the beginning of the lunar month (see [2]) form spherical data. The analysis of spherical data generally concentrates on the directional vector of the auditory object and, in most cases, ignores the distance effects. Under this assumption, the representation of the data reduces to a more tractable two-dimensional spherical display of the data namely latitude *θ* and longitude *φ*. While normal distribution is common for linear data, the von Mises-Fisher distribution is regularly considered for spherical data. The distribution is also known as Fisher distribution and assumes the data to be rotationally symmetric [3, 4].

Outliers are observations that are different in some way from the rest. For example, the wind direction on one particular day which is in the opposite direction to that observed on other days in the same monsoon season is a candidate to be an outlier. The existence of outliers in circular data has been shown to affect parameter estimation and weaken the accuracy of forecast (see for example [5, 6] and warrants proper treatment in the early stage of data analysis. At present, several discordancy tests are developed to detect outlier in 2-dimensional directional data including [7–10]. Fewer similar studies are conducted for spherical data [11]. Used probability plot as part of a preliminary examination on a given spherical data set to detect outlier. On the other hand [4], proposed formal tests of discordancy by extending the idea used in [5] for circular data. In this paper, we propose a new outlier detection method for spherical data using the *k*-nearest neighbours distance on a unit sphere. The distance between two points on the surface of a sphere is measured using the law of cosine. The proposed method can detect not only single and multiple outliers but also a patch of outliers.

This paper is organized as follows: Section 2 reviews two existing tests of discordancy in the Fisher distribution. Section 3 shows the distance between two-unit vectors. Section 4 reviews the definition of *k*-nearest neighbours distance. Section 5 presents a new test of discordancy for a patch of outliers. Through simulations, we obtain the percentage points of the test statistic and study its performance in Section 6. For illustration, an application of the methods on a real data set is presented in Section 7.

### Tests of discordancy in the fisher distribution

Fisher distribution is a common unimodal distribution considered for spherical data. The probability density functions of a Fisher distribution for a given random vector (*Θ*, *Φ*) is given by

f(θ,φ)=[κ/(4πsinhκ)]exp[κ{cosαcosθ+sinαsinθcos(φ−β)}]sinθ (1)

where 0≤*θ*, *α*<*π*; 0≤*φ*, *β*<2*π*; *κ*>0, (*α*, *β*) is the mean direction, and *κ* is a measure of the concentration about the mean direction.

Let (*θ*_1_, *φ*_1_),…,(*θ*_*n*_, *φ*_*n*_) be a random sample from a Fisher distribution with mean direction (*α*, *β*). Let $\left(\bar{\theta },\bar{\varphi }\right)$ be the sample mean direction, *R* be the sample resultant length given by

$$R=\sqrt{S_{x}^{2}+S_{y}^{2}+S_{z}^{2}}$$

where $S_{x}=\sum_{i=1}^{n}x_{i}$, $S_{y}=\sum_{i=1}^{n}y_{i},\;S_{z}=\sum_{i=1}^{n}z_{i}$, *x*_*i*_ = sin *θ*_*i*_ cos *φ*_*i*_, *y*_*i*_ = sin *θ*_*i*_ sin *φ*_*i*_, *z*_*i*_ = cos *θ*_*i*_ and $\bar{R}=R/n$ be the mean resultant length. Note that (*x*_*i*_, *y*_*i*_, *z*_*i*_) is in a direction of cosine. Further, $R_{n-1}^{\left(-i\right)}$ and ${\bar{R}}_{n-1}^{\left(-i\right)}$ denote the values of resultant length and mean resultant length, respectively, with the observation (*θ*_*i*_, *φ*_*i*_) omitted from the data set [4]. Recommended two test statistics, the *C*^*k*^ and *E*^*k*^ statistics. The analogue of Collett’s *C* statistic is defined as

$$C^{k}=\underset{i}{\max}\left\{\frac{{\bar{R}}_{n-1}^{\left(-i\right)}-\bar{R}}{\bar{R}}\right\},\;where\;i=1,2,\ldots ,n.$$ (2)

While the analogue of Collett’s *M* statistic is

$$E^{k}=(n-2)\left\{\frac{1+R_{n-1}^{\left(-i\right)}-R_{n}}{n-1-R_{n-1}^{\left(-i\right)}}\right\},\;where\;i=1,2,\ldots ,n.$$ (3)

[4] noted that the *C*^*k*^ statistic is a good statistic when *κ* is known or a good estimate if it is available. In addition, the *E*^*k*^ statistic is developed by considering intuitive and formal likelihood-ratio, whose distribution is available in compact form, and is independent of the value of *κ*. The *E*^*k*^ statistic is based on a generalized likelihood-ratio test against the alternative hypothesis that one observation is drawn from a Fisher distribution with different mean direction but the same concentration parameter. In addition, both test statistics can detect a single outlier and several outliers (see [4]).

## The distance on a sphere

For any 3-dimensional data set, we can find a distance of any given point on a sphere by calculating the distance between two vectors. The distance between two-unit vectors ***x***_**1**_ and ***x***_**2**_ (where both have a length of unit radius) can be calculated by using the law of cosine. Let *θ*_12_ be the angle between unit vectors ***x***_**1**_ = (*x*_1_, *y*_1_, *z*_1_) and ***x***_**2**_ = (*x*_2_, *y*_2_, *z*_2_). We can obtain the distance between the two points on a sphere by

$$\begin{array}{l} d(x_{1},x_{2})=(x_{1}-x_{2})•(x_{1}-x_{2})\\ \;={\left\|x_{1}\right\|}^{2}+{\left\|x_{2}\right\|}^{2}-2(\|x_{1}\|\|x_{2}\|\cos\;\theta_{12}). \end{array}$$ (4)

Therefore Eq (4) can be simplified to

$$d(x_{1},x_{2})=2-2\;\cos\;\theta_{12}$$

where 0≤*θ*_12_≤*π*. It is known that ***x***_**1**_•***x***_**2**_ = ‖***x***_**1**_‖‖***x***_**2**_‖ cos *θ*_12_. Then

$$\cos\;\theta_{12}=\frac{x_{1}•x_{2}}{\left\|x_{1}\|\|x_{2}\right\|}.$$

Given that ***x***_**1**_ and ***x***_**2**_ are two-unit vectors, it must be that $cos\;\theta_{12}=x_{1}^{T}x_{2}$. In general, the spherical distance between two-unit vectors is given by

$$d(x_{i},x_{j})=2-2\;{x_{i}}^{T}x_{j}.$$

For simplicity, we may remove the constant giving

$$d(x_{1},x_{2})=1-\cos\;\theta_{12}.$$ (5)

## The *K*-nearest neighbours distance

If *k* = 1, we consider the distance of first nearest neighbours for a given point, say ***x***_***i***_. First, we denote *d*_1*i*_(***x***_***i***_, ***x***_***j***_), for *j* = 1,2,…,*n*, *i*≠*j* as the distance of first nearest neighbours between the *i-*th observation and the rest of observations while *d*_(1*i*)_(***x***_***i***_, ***x***_***j***_) the corresponding ordered distances. The first-nearest distance for the *i-*th observation is then given by

$$Q_{i}^{1}=d_{\left(1i\right)}(x_{i},x_{j})\;\mathrm{for}\;j=1,2,...,n,i\neq j.$$ (6)

Note that $\left\{Q_{i}^{1},\;i=1,2,\ldots ,n\right\}$ gives a sequence of distances between successive observations on the *p*-dimensional surface. The statistic (6) can be generalized to detect a patch of outliers in spherical data by calculating the *k*-nearest distance for the *i-*th observation. For that, we define $Q_{i}^{k}$ as the *k*-nearest neighbours distance for the *i*th ordered observation, *k* = 1,2,3,… and *i* = 1,2,…,*n* such that

$$Q_{i}^{k}=d_{\left(ki\right)}(x_{i},x_{j})\;for\;j=1,2,\ldots ,n,i\neq j.$$ (7)

We will use the statistic (7) in the development of a new method for detecting a single, multiple as well as a patch of outliers in the following section.

### A new method of outlier detection for spherical data

In this section, we use the *k*-nearest neighbours distance as a basic idea to be used in the development of a new method to detect possible outliers in spherical data, denoted by *Q*^*k*^. Suppose *x*_1_, *x*_2_,…,*x*_*n*_ are (*i*.*i*.*d*) spherical observations from a Fisher distribution of sample size *n*. The sample vector of a spherical sample is given by *x*_*i*_ = (*x*_*i*_, *y*_*i*_, *z*_*i*_). Thus, the procedure to obtain the outlier detection method using the *Q*^*k*^ statistic is described as follows:

Step 1 Start with *k* = 1. Calculate $Q_{i}^{1}$, *i* = 1,2,…,*n* as given by Eq (7).
Step 2 If the value of $Q_{i}^{1}$ exceeds a pre-determined cut-off point, say *C*_*Q*_, then the *i*-th observation corresponding to $Q_{i}^{1}$ is identified as an outlier and the process is stopped. Otherwise, proceed to the next step.
Step 3 Increase *k* by one, that is, *k* = 2. Calculate $Q_{i}^{2}$, *i* = 1,2,…,*n*.
Step 4 If the value of $Q_{i}^{2}$ exceeds a pre-determined cut-off point, say *C*_*Q*_, then the observations corresponding to $Q_{i}^{2}$ are identified as a patch of two outliers and the process is stopped. Otherwise, the process continues by increasing the value of *k* by one at a time in the subsequence steps.

First, we need to obtain the cut-off points *C*_*Q*_ for the *Q*^*k*^ statistic. We design a simulation study using the R software to find the percentage points under the null hypothesis of no outliers in the circular data set. Note that parameters *α* and *β* are spherical location parameters while *κ* is a concentration parameter. We found that the distances between observations generated from a Fisher distribution depend on *n* and *κ* but not on *α* and *β* (the detail is not given here). For each combination of *n* and *κ*, we generate a sample from Fisher distribution with both location parameters fixed (*α* = 0, *β* = 0) and calculate the *Q*^*k*^ statistic. Then, we repeat the process 3000 times and estimate the percentage points of the *Q*^*k*^ statistic at 10%, 5% and 1% upper percentiles when no outlier is present in the sample. Selected cut-off points *C*_*Q*_ for the *Q*^*k*^ statistic are tabulated in Tables 1–3 for *k* = 1, 2 and 3 respectively.

**Table 1:** Cut-off points, *C*_*Q*_ for *Q*^1^ statistic.

	Level of percentiles	*κ*									
*n*		2	3	4	5	7	10	20	30	40	50
10	10%	0.89	0.74	0.58	0.45	0.33	0.23	0.11	0.08	0.06	0.05
	5%	1.01	0.90	0.71	0.56	0.41	0.29	0.14	0.09	0.07	0.06
	1%	1.23	1.16	0.99	0.81	0.58	0.46	0.20	0.13	0.09	0.09
30	10%	0.61	0.61	0.47	0.40	0.25	0.18	0.09	0.06	0.04	0.04
	5%	0.70	0.75	0.60	0.49	0.31	0.22	0.11	0.07	0.05	0.04
	1%	0.88	0.97	0.97	0.72	0.48	0.33	0.17	0.11	0.08	0.06
50	10%	0.51	0.56	0.45	0.35	0.23	0.16	0.08	0.05	0.04	0.03
	5%	0.57	0.67	0.57	0.47	0.30	0.20	0.10	0.06	0.05	0.04
	1%	0.74	0.86	0.85	0.68	0.44	0.33	0.14	0.09	0.07	0.06
80	10%	0.41	0.49	0.44	0.34	0.23	0.16	0.07	0.05	0.04	0.03
	5%	0.47	0.60	0.54	0.45	0.30	0.20	0.09	0.06	0.04	0.03
	1%	0.61	0.80	0.76	0.76	0.50	0.31	0.14	0.09	0.07	0.05
100	10%	0.36	0.48	0.44	0.34	0.22	0.15	0.07	0.05	0.03	0.03
	5%	0.41	0.56	0.54	0.44	0.29	0.19	0.09	0.06	0.04	0.03
	1%	0.53	0.76	0.78	0.72	0.42	0.31	0.13	0.08	0.06	0.05
200	10%	0.26	0.40	0.40	0.32	0.21	0.14	0.06	0.04	0.03	0.03
	5%	0.30	0.47	0.49	0.41	0.26	0.17	0.08	0.05	0.04	0.03
	1%	0.37	0.61	0.69	0.65	0.42	0.24	0.13	0.08	0.06	0.05

**Table 2:** Cut-off points, *C*_*Q*_ for *Q*^2^ statistic.

	Level of percentiles	*κ*									
*n*		2	3	4	5	7	10	20	30	40	50
10	10%	1.16	1.00	0.77	0.64	0.46	0.31	0.16	0.11	0.08	0.06
	5%	1.27	1.17	0.92	0.75	0.54	0.37	0.20	0.13	0.10	0.08
	1%	1.47	1.44	1.27	1.00	0.77	0.51	0.27	0.18	0.13	0.10
30	10%	0.80	0.79	0.63	0.51	0.35	0.24	0.12	0.08	0.06	0.05
	5%	0.88	0.94	0.78	0.62	0.41	0.29	0.14	0.09	0.07	0.06
	1%	1.04	1.18	1.10	0.88	0.57	0.42	0.20	0.13	0.11	0.08
50	10%	0.66	0.74	0.63	0.48	0.32	0.22	0.11	0.07	0.05	0.04
	5%	0.75	0.84	0.75	0.57	0.39	0.26	0.13	0.08	0.06	0.05
	1%	0.89	1.03	1.01	0.80	0.59	0.36	0.19	0.12	0.09	0.07
80	10%	0.54	0.65	0.56	0.44	0.29	0.20	0.10	0.07	0.05	0.04
	5%	0.59	0.74	0.67	0.56	0.37	0.25	0.11	0.08	0.06	0.05
	1%	0.72	0.90	0.93	0.82	0.53	0.34	0.16	0.11	0.08	0.07
100	10%	0.50	0.63	0.58	0.44	0.29	0.19	0.09	0.06	0.05	0.04
	5%	0.56	0.72	0.69	0.55	0.35	0.22	0.11	0.07	0.05	0.04
	1%	0.66	0.89	0.96	0.77	0.54	0.34	0.16	0.11	0.08	0.06
200	10%	0.41	0.56	0.55	0.44	0.28	0.19	0.09	0.06	0.04	0.03
	5%	0.46	0.64	0.67	0.55	0.36	0.23	0.11	0.07	0.05	0.04
	1%	0.57	0.81	0.91	0.85	0.52	0.34	0.16	0.10	0.07	0.05

**Table 3:** Cut-off points, *C*_*Q*_ for *Q*^3^ statistic.

	Level of percentiles	*κ*									
*n*		2	3	4	5	7	10	20	30	40	50
10	10%	1.33	1.18	0.93	0.77	0.54	0.39	0.20	0.13	0.10	0.08
	5%	1.43	1.33	1.08	0.93	0.63	0.46	0.24	0.15	0.12	0.10
	1%	1.61	1.59	1.42	1.25	0.85	0.61	0.33	0.21	0.16	0.13
30	10%	0.94	0.92	0.77	0.60	0.42	0.28	0.14	0.10	0.07	0.05
	5%	1.04	1.05	0.91	0.71	0.51	0.34	0.17	0.11	0.08	0.06
	1%	1.23	1.28	1.21	1.01	0.69	0.48	0.23	0.15	0.12	0.09
50	10%	0.79	0.83	0.71	0.52	0.38	0.26	0.13	0.08	0.06	0.05
	5%	0.86	0.96	0.83	0.64	0.44	0.31	0.15	0.10	0.07	0.06
	1%	1.01	1.14	1.14	0.99	0.59	0.43	0.21	0.13	0.10	0.08
80	10%	0.65	0.78	0.65	0.53	0.35	0.24	0.11	0.07	0.06	0.04
	5%	0.71	0.88	0.79	0.66	0.42	0.29	0.14	0.09	0.07	0.06
	1%	0.83	1.02	1.05	0.94	0.62	0.40	0.19	0.12	0.09	0.08
100	10%	0.59	0.73	0.68	0.50	0.34	0.24	0.11	0.07	0.06	0.04
	5%	0.65	0.81	0.82	0.61	0.41	0.29	0.13	0.09	0.06	0.05
	1%	0.75	0.96	1.01	0.93	0.61	0.38	0.19	0.13	0.09	0.07
200	10%	0.49	0.67	0.64	0.49	0.33	0.22	0.10	0.07	0.05	0.04
	5%	0.54	0.74	0.75	0.60	0.41	0.27	0.12	0.08	0.06	0.05
	1%	0.62	0.86	0.96	0.81	0.61	0.36	0.18	0.11	0.09	0.07

For most combinations of the concentration parameter *κ* and percentile level, the cut-off point decreases as the sample size increases. It can also be seen that, for small sample sizes, the cut-off points are a decreasing function of *κ*. For larger sample sizes, the cut-off points have a peak value at around *κ* = 30. The results indicate the proposed statistic depends on *n* and *κ* of the underlying assumed model. As one might expect, it is also noted that the cut-off point also increases as the value of the *k*-nearest distance increases due to larger distances on the sphere between the points of interest.

### The performance of the *Q*^*k*^ statistic

Let *P*5 be the probability that the contaminant point is an outlying point and is identified as discordance [12, p.185] and [13, p.64-68]. Stated that a good test is expected to have a high *P*5 [4]. Investigated the performance of several methods to detect a single outlier in spherical distribution. Therefore, we compare the performance of the *Q*^*k*^ statistic with the existing methods of *E*^*k*^ and *C*^*k*^ statistics to detect a single outlier and a patch of outliers for various values of sample size and concentration parameters.

To study the performance of the *Q*^*k*^, *E*^*k*^ and *C*^*k*^ statistics to detect a single outlier, we first generate samples for two cases, a) *n* = 10, *κ* = 3 and b) *n* = 30, *κ* = 50. The samples are generated in such a way that *n*−1 of the observations come from Fisher distribution with *α* = 0, *β* = 0 while one observation (outlier) from Fisher distribution with *α* = *λπ*, *β* = 0, *κ* = 30, 0≤*λ*≤1. If the value of $Q_{i}^{k}$, $E_{i}^{k}$ and $C_{i}^{k}$ are greater than the corresponding cut-off point and the *i*^th^ observation is located at the outlying value, then we have correctly detected an outlier. We repeat the simulation 3000 times and obtain the value of *P*5 or known as probability of correct detection of an outlier which has been introduced into the samples. Note that, the cut-off points for the *E*^*k*^ and *C*^*k*^ statistics are obtained from Monte Carlo simulation according to the procedure in obtaining the cut-off points for *Q*^*k*^ statistic.

**Fig 1:**
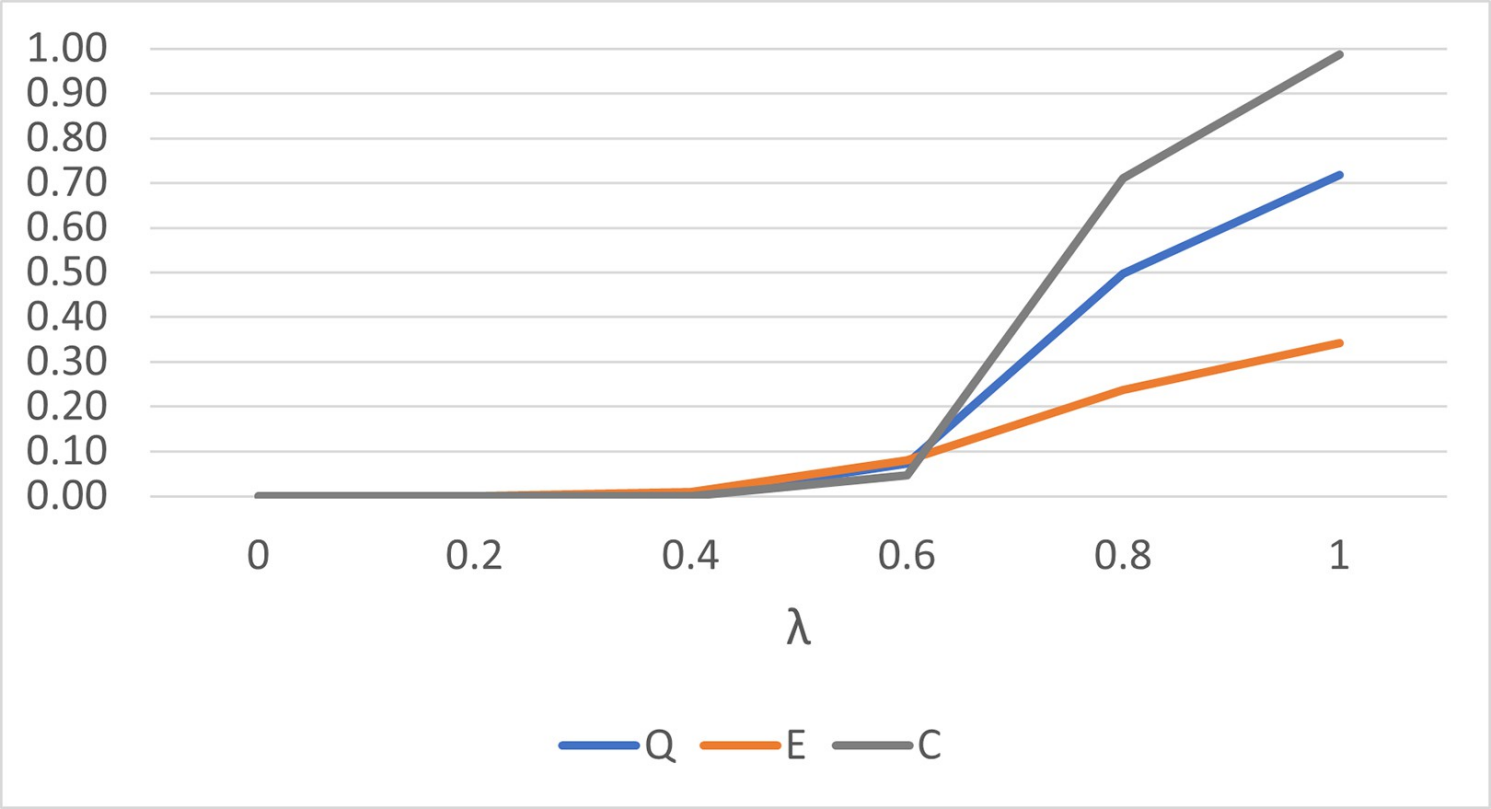
The performance of the *Q*^*k*^, *E*^*k*^ and *C*^*k*^ statistics for *n* = 10 and *κ* = 3 for a single outlier.

**Fig 2:**
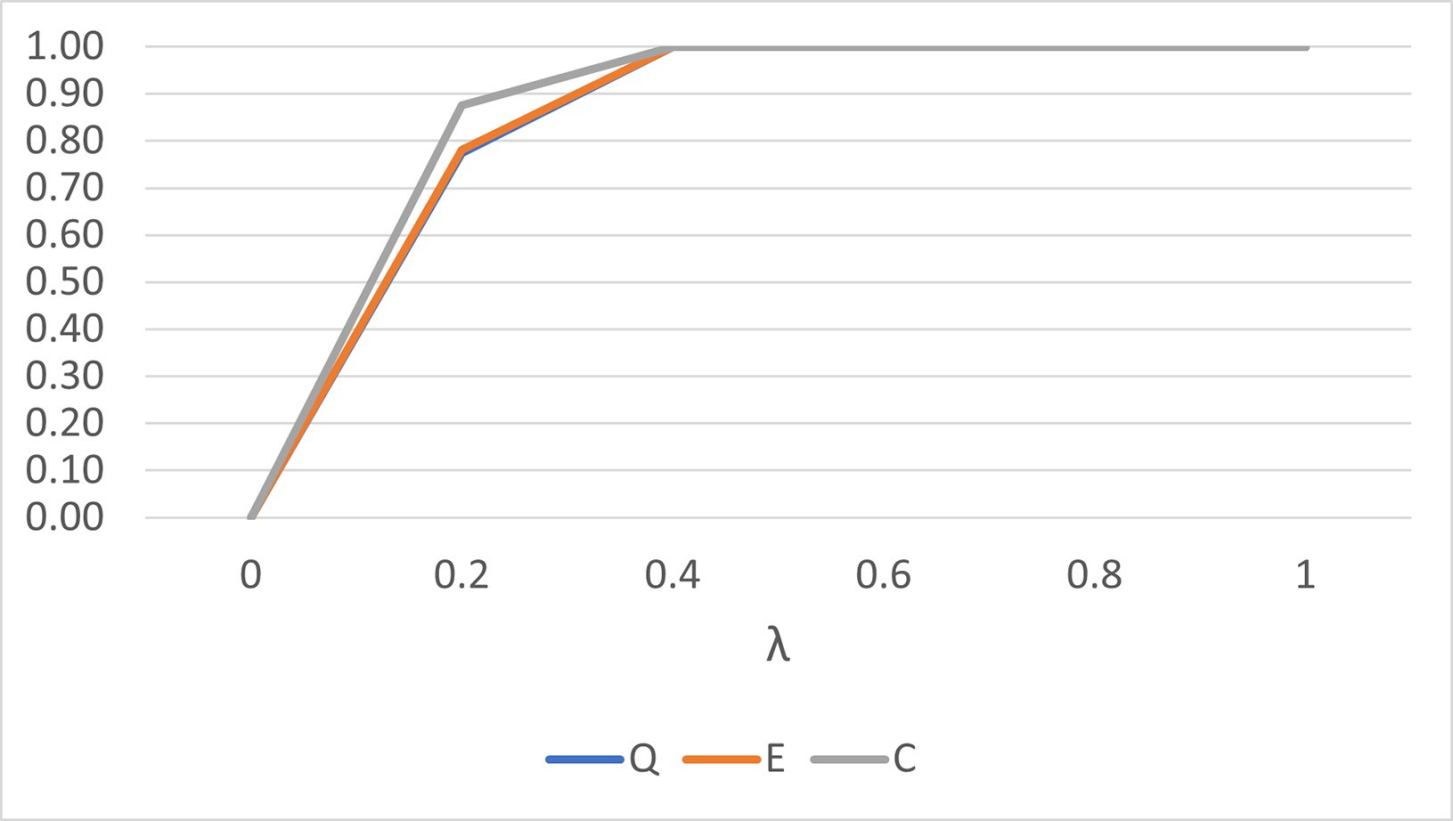
The performance of the *Q*^*k*^, *E*^*k*^ and *C*^*k*^ statistics for *n* = 30 and *κ* = 50 for a single outlier.

Fig 1 plots the performance of the *Q*^*k*^, *E*^*k*^ and *C*^*k*^ statistics to detect a single outlier for small sample size and small concentration parameter value. Generally, for small sample size, the *Q*^*k*^ statistic performs better than the *E*^*k*^ statistic only. However, the performance is almost identical when larger sample size and larger concentration parameter values are considered as shown in Fig 2.

**Fig 3:**
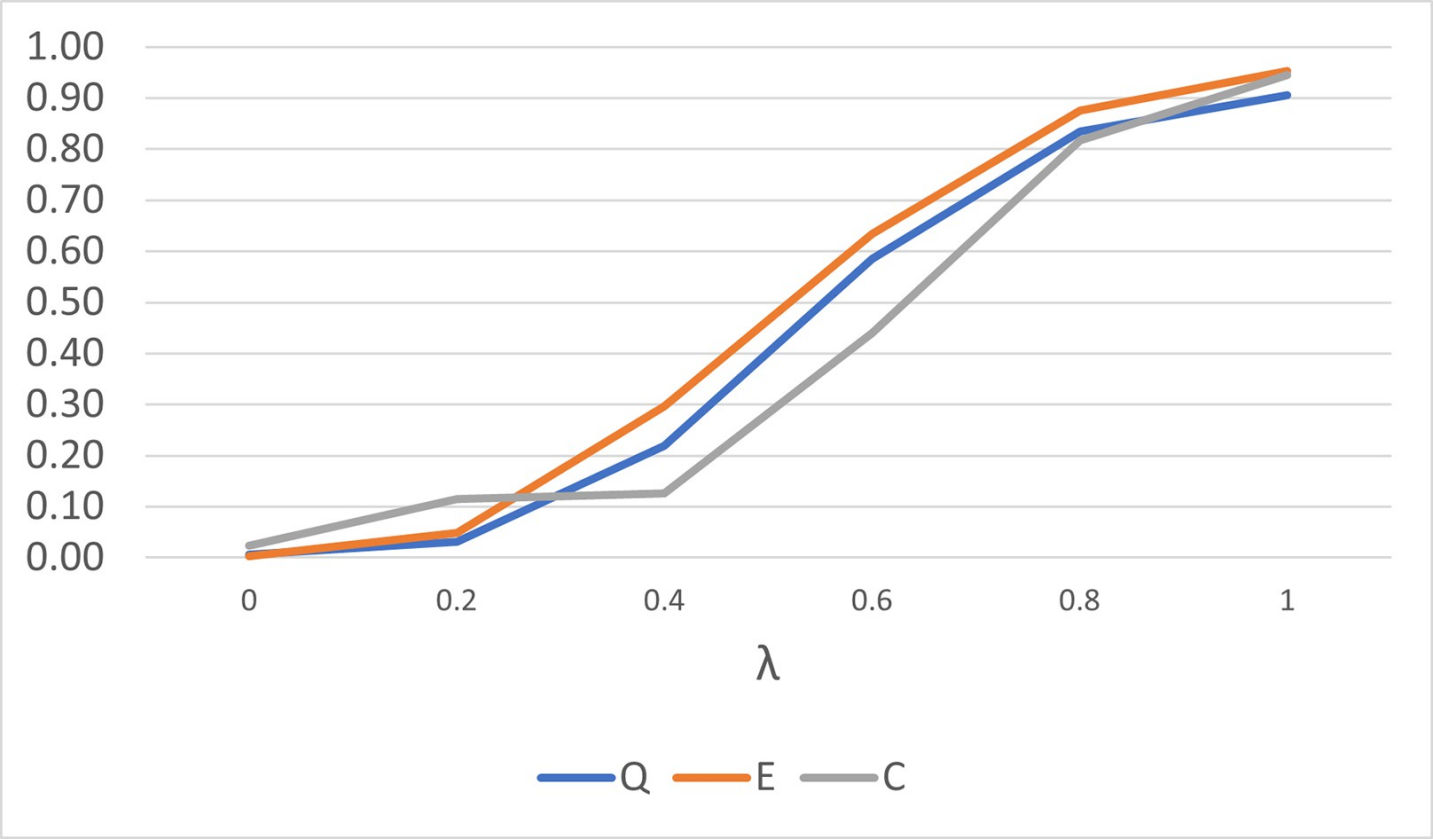
The performance of the *Q*^*k*^, *E*^*k*^ and *C*^*k*^ statistics for *n* = 10 and *κ* = 3, for a patch of three outliers.

**Fig 4:**
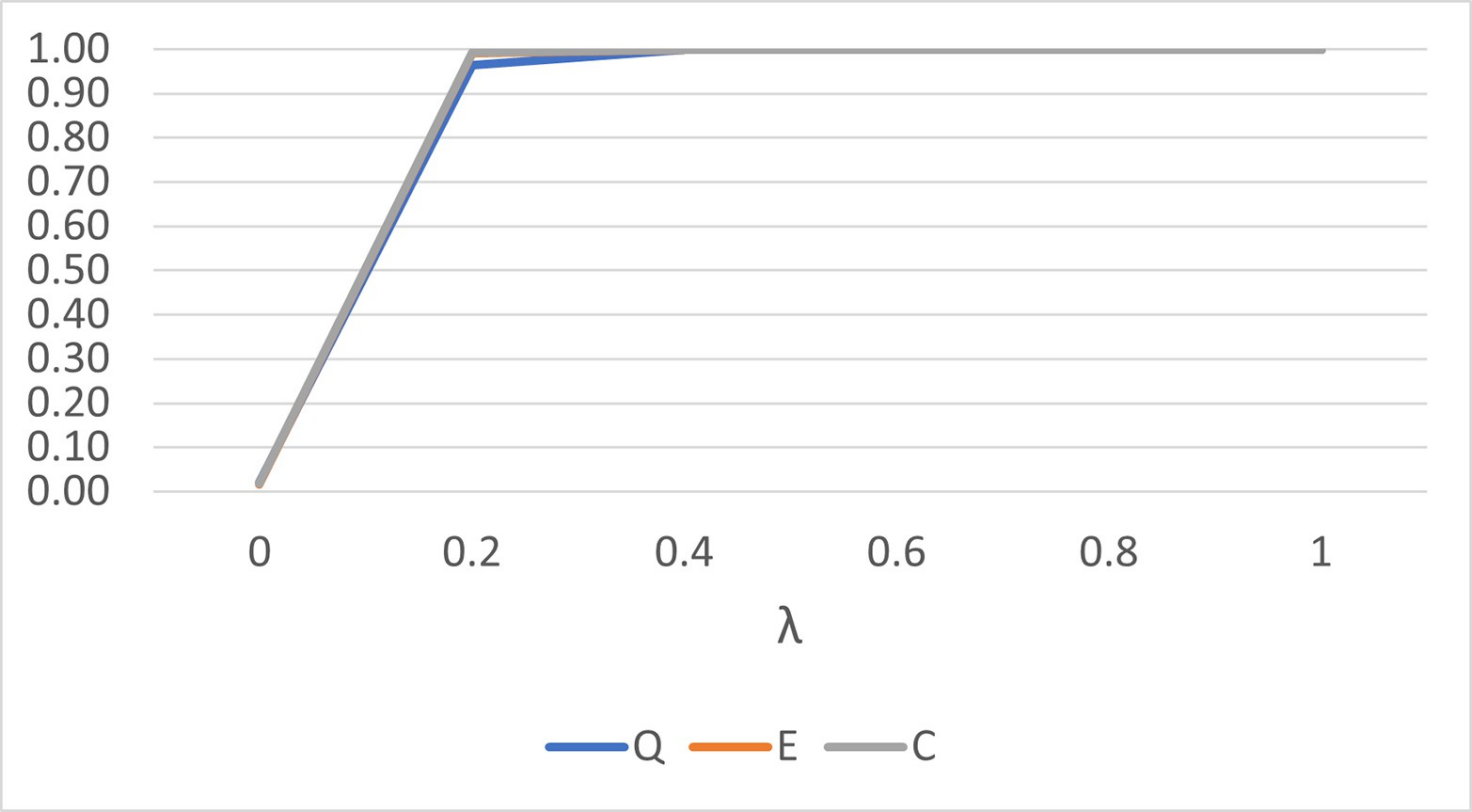
The performance of the *Q*^*k*^, *E*^*k*^ and *C*^*k*^ statistics for *n* = 30 and *κ* = 50, for a patch of three outliers.

We also investigate the performance to detect a patch of outliers for the three statistics. For small sample size and small concentration parameter value, the performance of the *Q*^*k*^ statistic is comparable to the *E*^*k*^ and *C*^*k*^ statistics as shown in Fig 3. A much closer result is observed for larger sample size and larger concentration values as shown in Fig 4. The trend is observed for other combinations of sample size and concentration parameter values. This suggests that the *Q*^*k*^ statistic can be a good alternative outlier detection method for spherical data.

## Practical example

For illustration, we now apply the proposed and existing spherical discordancy tests into a set of eye data. We consider the eye data consisting of 23 patients (unit in radians) recorded using optical coherence tomography (OCT) at the University Malaya Medical Centre (UMMC). OCT technology originally is used in ophthalmology to image the posterior segment and has also been used to image anterior segment structures such as the cornea. The angle imaging of the anterior segment OCT in UMMC patients’ eyes were obtained with Anterior Segment OCT (AS-OCT). The measurements selected are the angle of the posterior corneal curvature, *φ*, and the angle of the eye (between posterior corneal curvature to iris), *θ*. As such, we are keen to identify possible outliers in this data set as given in Table 4.

**Table 4:** The bivariate eye data.

Patient	*φ* (rad)	*θ* (rad)	Patient	*φ* (rad)	*θ* (rad)
1	1.599	0.422	13	1.470	0.981
2	1.208	0.463	14	1.744	1.023
3	1.456	0.733	15	1.674	1.286
4	2.098	0.733	16	1.382	0.937
5	1.401	0.684	17	0.557	0.909
6	1.819	0.944	18	1.688	0.642
7	1.569	0.757	19	1.628	0.724
8	1.562	0.705	20	1.560	0.656
9	1.850	0.632	21	1.808	0.646
10	0.639	0.644	22	2.089	0.471
11	1.696	0.930	23	2.293	0.154
12	1.965	0.429			

**Fig 5:**
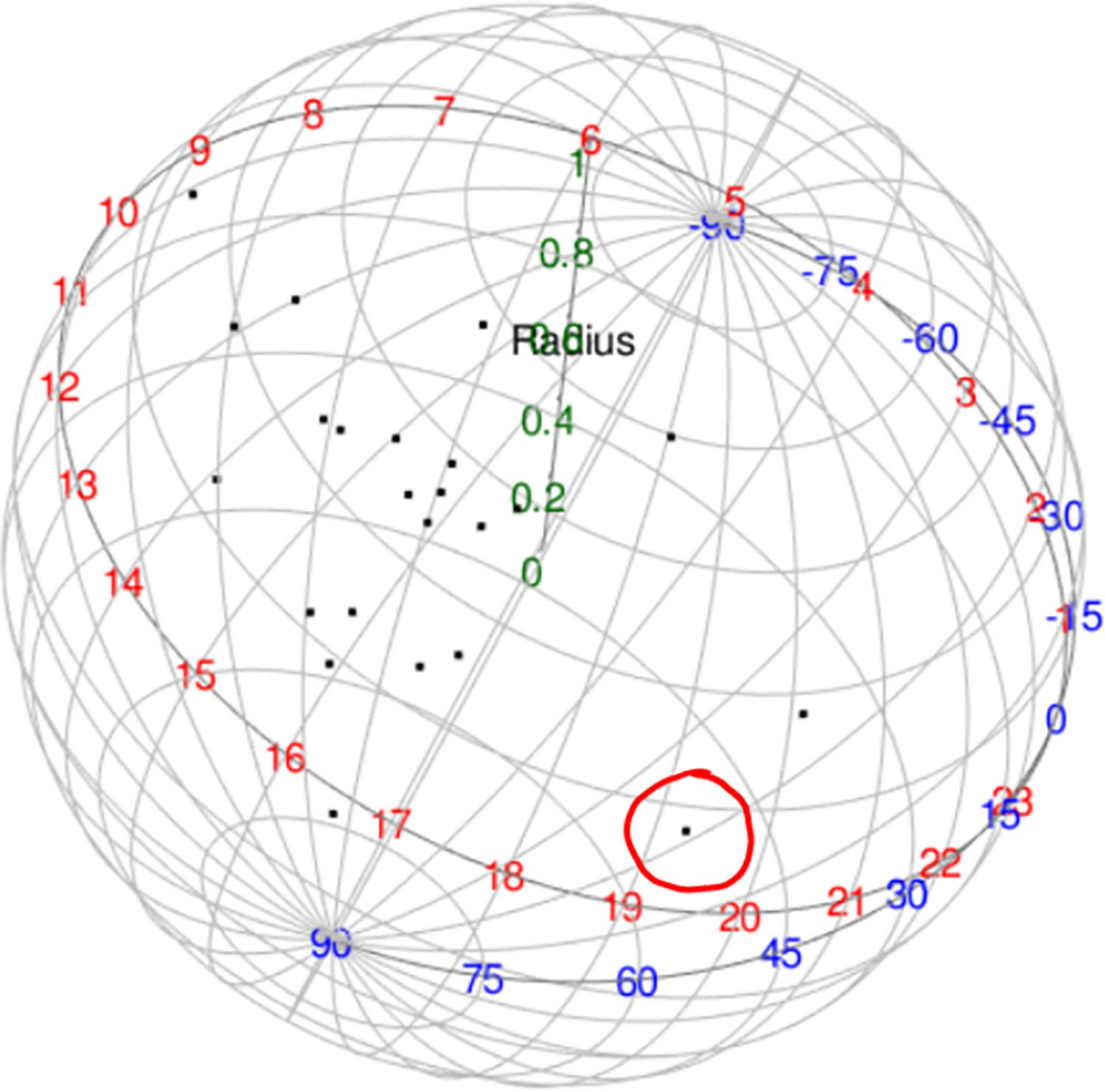
Spherical plot of eye data.

The summary statistics for the given spherical data set are calculated; the sample mean direction is given in a longitude and latitude expression, $\left(\hat{\theta }=0.6833,\;\hat{\varphi }=1.5744\right)$ with the concentration parameter $\hat{\kappa }=17.9100$. The spherical plot of the data is given in Fig 5. The samples are located around a north pole. This indicates that both variables, namely the posterior and angle of the eye, recorded these 23 observations to be in the same direction. However, there is one observation lying further away from the rest.

It is known that Q-Q plot and probability plotting are commonly used to investigate the goodness of fit of linear, circular and spherical data samples (see for example [14–16]). It is used to visualize the goodness of fit and to identify the presence of outlier(s) at earlier stage [11]. Provided procedures of plotting an ordered value for spherical data which is assumed to follow a Fisher distribution. They proposed three types of procedures, namely, colatitudes, longitude and two-variable plotting procedure for a Fisher model. The procedures considered three-ordered-value plots. Two of them examine the marginal distributions of the two variables and one of them is to find the association between these two variables. The details of the procedures can be obtained in [11]. Note that, the quantile of the unit exponential distribution is denoted by *e*, $U_{i,n}=\frac{\left(i-\frac{1}{2}\right)}{n}$ for the uniform model is denoted by *u* and the quantile of the *N*(0,1) distribution is denoted by *q*.

**Fig 6:**
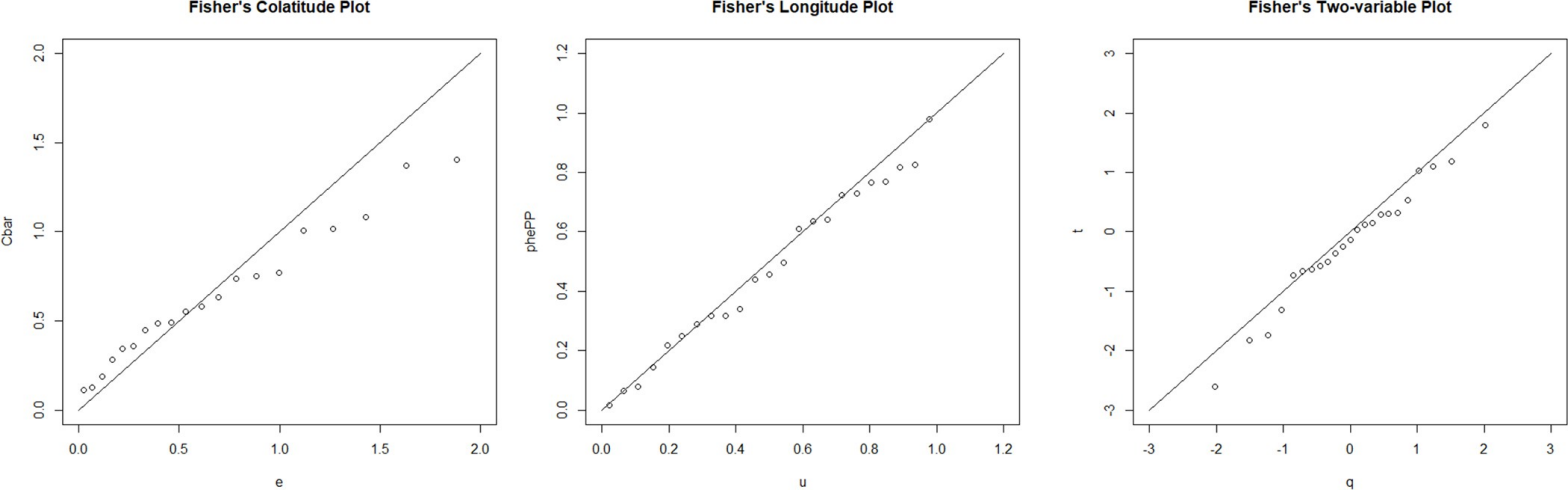
Plots of eye data, (a) Colatitude plot, (b) Longitude plot, (c) Two-variable plot.

The colatitude plot of the eye data as shown in Fig 6(A) indicates that the data follow Fisher distribution as the plot gives almost a straight line through the origin. This is further supported by the longitude plot as shown in Fig 6(B) which gives an approximately straight linear plot of slope close to 45° passing through the origin. From Fig 6(C), we can clearly see one observation that lies far from the rest, indicating the existence of one possible outlier. Therefore, we apply the proposed discordancy test on the data. Upon applying the maximum likelihood estimation method, we obtain the estimate parameters of the Fisher distribution. The values of the parameters are $\hat{\alpha }=0.6833$ and $\hat{\beta }=1.5744$.

Based on the estimated parameters, we obtain the critical values of three test statistics using the R statistical software. The values are shown in Table 5. We apply the discordancy tests including our proposed test statistic and obtain their test statistic values. The values of the test statistics which correspond to observation number 17 are $C_{17}^{k}=0.0104$, $E_{17}^{k}=5.6622$, $Q_{17}^{1}=0.0366$, $Q_{17}^{2}=0.1702$ and $Q_{17}^{3}=0.1930$. Table 5 shows the cut-off points for the three methods at 10% significance levels. Based on Table 5, only *Q*^2^ and *Q*^3^ statistics can detect observation 17 as an outlier at 10% upper level. This observation corresponds to a patient with small values of angle of the posterior corneal curvature compared to other patients and thus may warrant further investigation.

**Table 5:** The (10% upper level) critical values of discordancy tests for *n* = 23 and *κ* = 17.9100.

Statistics	*C* ^*k*^	*E* ^*k*^	*Q* ^1^	*Q* ^2^	*Q* ^3^
Critical values	0.0116	6.2700	0.1015	0.1393	0.1659

Next, we are keen to demonstrate the application of the tests to detect a patch of outliers. Observation 10 is chosen and located closely to observation 17 so that a patch of two outliers exist in the data. The new coordinate for observation 10 is *θ* = 0.9599, *φ* = 0.6109.

**Fig 7:**
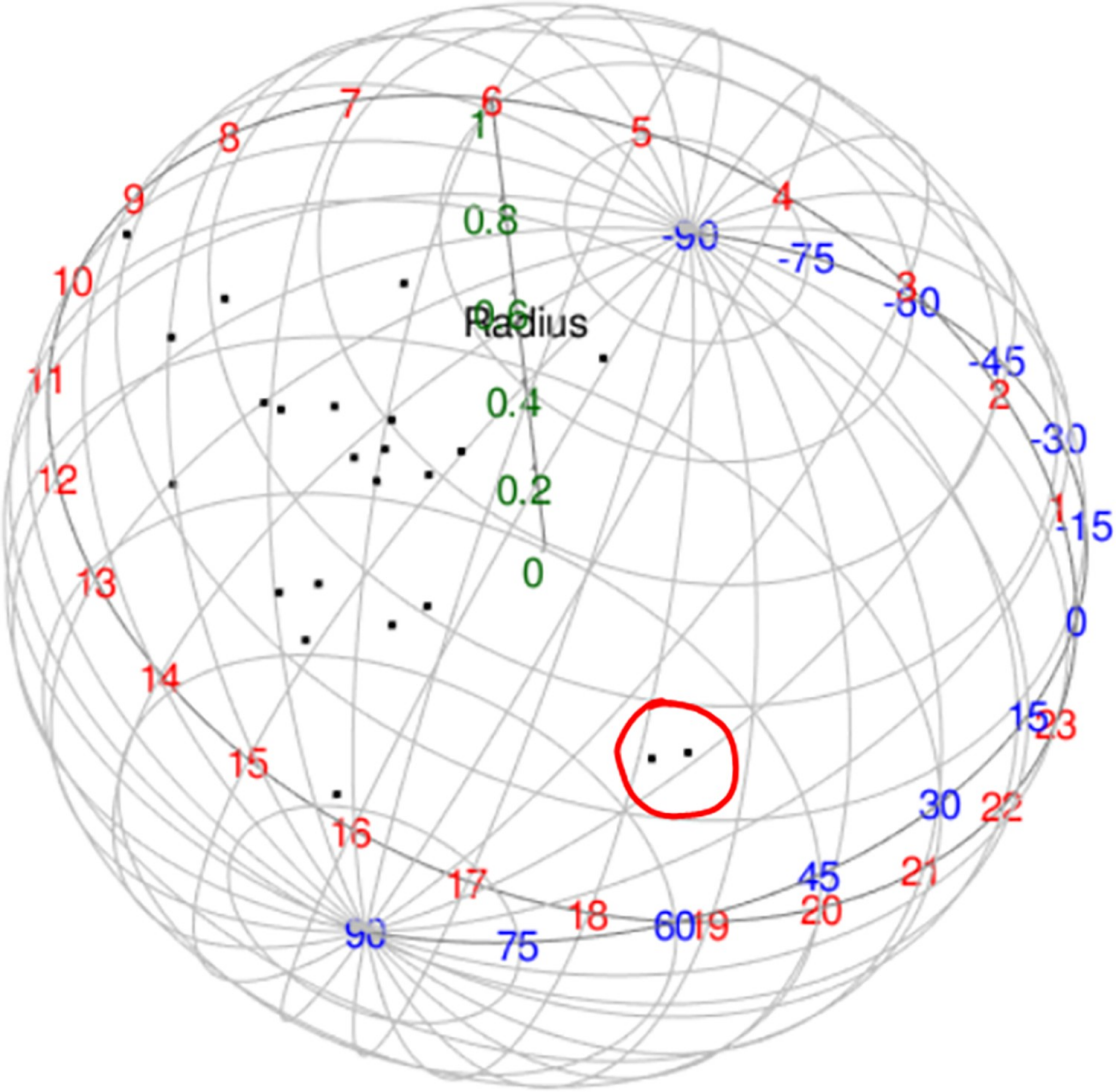
Spherical plot of eye data (a patch of two outliers).

From Fig 7, it can be seen clearly that both observations (observations 10 and 17) are located far from the rest. Upon applying descriptive statistics, the value of the sample mean direction is given in a longitude and latitude expression, $\left(\hat{\theta }=0.6939,\;\hat{\varphi }=1.5607\right)$ and the concentration parameter $\hat{\kappa }=16.5789$. The values of the test statistics and the cut-off points for the three methods at 10% significance levels are given in Table 6. As a result, the *Q*^2^ and *Q*^3^ statistics successfully detected observations 10 and 17 as a patch of two outliers at 10% upper level while the other test statistics failed.

**Table 6:** The test statistics values and the (10% upper level) critical values of discordancy tests for *n* = 23 and *κ* = 16.5789.

Statistics	*C* ^*k*^	*E* ^*k*^	*Q* ^1^	*Q* ^2^	*Q* ^3^
Critical values	0.0122	6.1296	0.1114	0.1473	0.1755
Observation 10	0.0099	4.9557	0.0022	0.1844	0.1871
Observation 17	0.0100	5.0162	0.0022	0.1702	0.1928

## Conclusion

In this paper, we proposed a new discordancy test for detecting outliers in spherical data based on the *k*-nearest neighbours distance. We further demonstrated the applicability of the proposed *Q*^*k*^ statistic on the eye data set by successfully identifying a single outlier and a patch of outliers in the data. A novel aspect of this method is in its ability to detect a patch of outliers which can be enhanced for cluster analysis in spherical data. The proposed procedure should work for other spherical distributions.
